# A comparative study on the application of different endoscopic diagnostic methods in the differential diagnosis of benign and malignant bile duct strictures

**DOI:** 10.3389/fmed.2023.1143978

**Published:** 2023-07-13

**Authors:** Liang Zhu, Zhi-Quan Huang, Zhen-Wen Wang, Xue-Ping Yang, Jun-Bo Hong, Zhen-Zhen Yang, Zheng-Ping Yu, Rong-Lai Cao, Jin-Li He, You-Xiang Chen

**Affiliations:** ^1^Department of Gastroenterology, The First Affiliated Hospital of Nanchang University, Nanchang, China; ^2^Jiangxi Clinical Research Center for Gastroenterology, Nanchang, China

**Keywords:** bile duct stricture, endoscopic retrograde cholangiopancreatography (ERCP), Spyglass, differential diagnosis, safety

## Abstract

**Objective:**

To compare the diagnostic value of cytobrush, ERCP-guided biopsy, SpyGlass direct visual impression and SpyGlass-guided biospy (SpyBite) in the differential diagnosis of benign and malignant bile duct strictures.

**Methods:**

The data of 1,008 patients who were clinically diagnosed with indeterminate biliary strictures and underwent ERCP-guided biopsy, cytobrush, SpyGlass direct visual impression or SpyBite at the First Affiliated Hospital of Nanchang University between January 2010 and December 2019 were collected and analyzed retrospectively. The final diagnose was determined by surgical pathological specimen or follow-up (Malignant stricture can be identified if the stricture showed malignant progression during one year of follow-up). The differential diagnostic value of the above endoscopic diagnostic methods was evaluated by means of sensitivity, specificity, accuracy, positive predictive value, negative predictive value, etc. and safety was evaluated by the incidence rate of adverse events.

**Results:**

In terms of sensitivity, standard biopsy group (48.6%) and SpyBite group (61.5%) were significantly higher than cytobrush group (32.0%), and visual impression group (100%) was significantly higher than any other group. As far as specificity was concerned, cytobrush group (99.0%), standard biopsy group (99.3%) and the SpyBite group (100%) were significantly higher than visual impression (55.6%), but there was no statistical difference among the three groups above. As far as accuracy was concerned, standard biopsy group (65.3%), and SpyBite group (80.0%) were significantly higher than cytobrush group (44.4%), and SpyBite group (80.0%) was significantly higher than visual impression group (54.8%). In terms of safety, visual impression group and SpyBite group were significantly higher than cytobrush group and standard biopsy group in post-ERCP cholangitis.

**Conclusion:**

SpyBite combined with SpyGlass-guided visual impression was better for differential diagnosis of benign and malignant bile duct strictures in terms of sensitivity and accuracy compared with conventional endoscopic diagnostic methods such as cytobrush and standard biopsy. Furthmore, the incidence rates of adverse events after SpyGlass examination was similar to those after conventional endoscopic diagnostic methods except for higher cholangitis, which could be controlled by antibiotics and might be avoided by adequate biliary drainage.

## Introduction

The bile duct stricture is generally defined as a narrowing or occlusion of the duct lumen caused by various factors including benign disorders such as fibrotic thickening or scarring repair of the bile duct wall, and malignant tumors, causing clinical symptoms such as poor bile drainage, impaired liver function and jaundice, and therefore can be classified as benign and malignant bile duct stricture (1).

Benign and malignant biliary strictures have different prognosis and need different management (1). Benign biliary strictures (BBSs) have a much better prognosis than malignant biliary strictures (MBSs) and the endoscopic approach has become the first-line option for most cases of BBSs (1). However, surgery remains the mainstay of cure in early stage of MBSs, but unfortunately only a minority of patients is diagnosed with resectable MBSs and more patients are in the advanced stage of tumors and might have lost the opportunity for surgery, resulting in poor prognosis (2). In the last decade, a large number attempts have been made to impove the median overall survival of the patient with MBSs by systemic treatment including chemotherapy and immunotherapy and by local treatments including radiofrequency, chemoembolization and radio-embolization (2–4). However, patients with advanced MBSs often suffer from severe jaundice and biliary drainage by endoscopic retrograde cholangiopancreatography (ERCP) or percutaneous transhepatic cholangial drainage (PTCD) for relieving symptoms and improving life quality of the patients is a necessary premise for systemic chemotherapy and palliative radiotherapy (5).

Therefore, it is important to distinguish between benign and malignant bile duct strictures for assessing the prognosis of patients and choosing a reasonable treatment. However, BBSs and MBSs often have similar clinical manifestations and imaging characteristics at the early stage, so endoscopic acquisition of pathological specimen is extremely important for the differential diagnosis of benign and malignant biliary strictures. However, the current conventional methods to obtain pathological specimen of biliary duct including ERCP-guided biopsy and cytobrush examination are not effective enough for the differential diagnosis due to the low sensitivity (6). SpyGlass choledochoscope, developed by Boston Scientific, also known as a single operator choledochoscope (SOC), provides direct visualization of the bile duct with high resolution (7). Furthermore, SpyGlass choledochoscope can be used to perform biopsy on the target site with SpyGlass-guided biospy (SpyBite) biopsy forceps, which bring great convenience to explore indeterminate bile duct strictures (8).

Although several studies have compared the diagnostic values of the endoscopic diagnostic methods mentioned above (9–11), they had the shortcomings of small sample size, unclear diagnostic criteria for benign and malignant strictures or large disparity in results, and therefore cannot fully demonstrate whether SpyGlass examination is better than ERCP-guided biopsy or cytobrush examination. In this study, we compared the value of the endoscopic diagnostic methods mentioned above in the differential diagnosis of benign and malignant biliary stricture by analyzing data of over 1,000 patients who underwent endoscopic diagnosis in our hospital during 10 years, to further provide evidence of the appropriate diagnostic method for indeterminate biliary strictures.

## Materials and methods

### Patients selection

**Figure 1:**
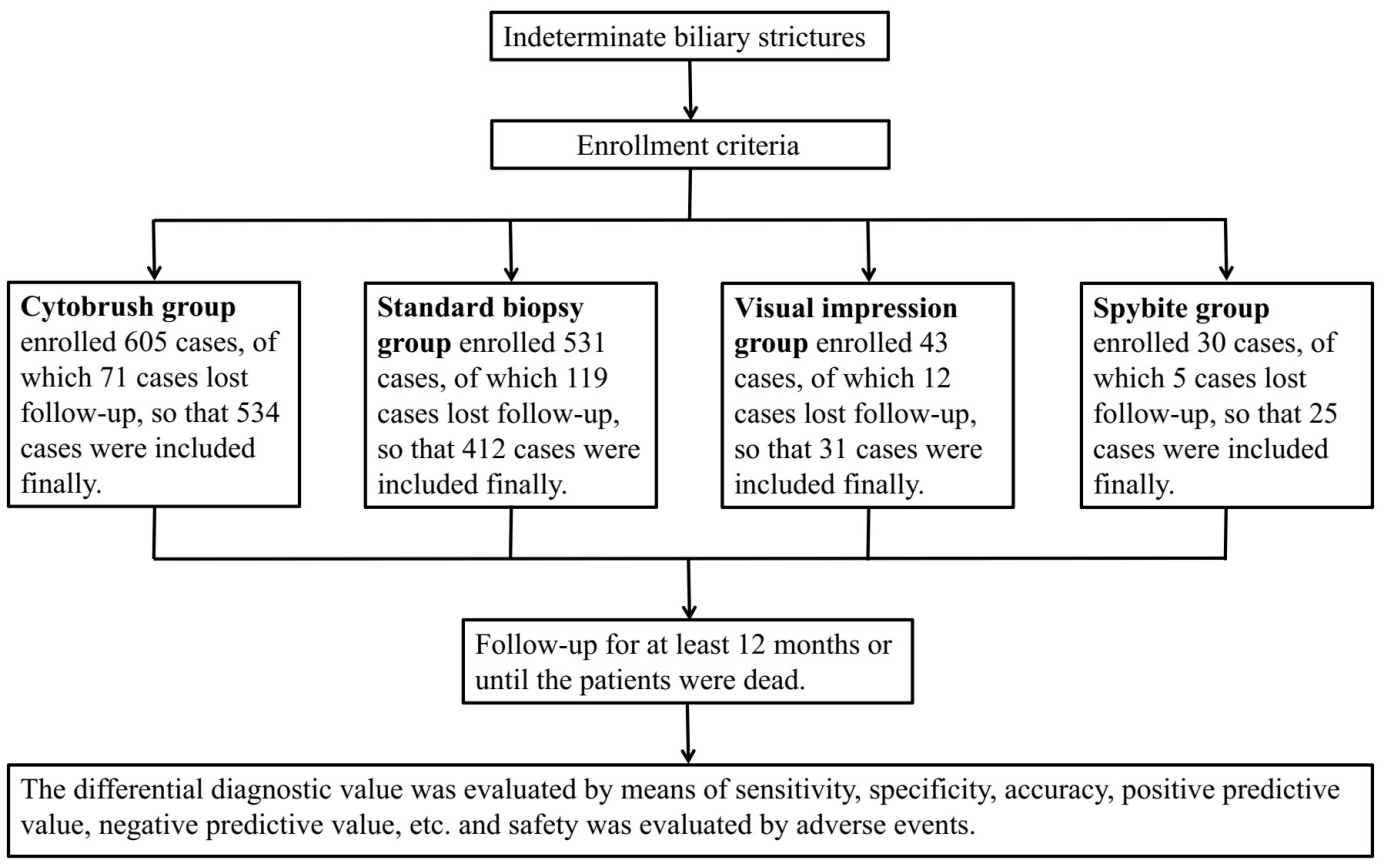
Flow diagram of patients enrollment.

This is a single-central retrospective study that finally included a total of 1,008 patients who were clinically diagnosed with indeterminate biliary strictures and underwent ERCP-guided biopsy, cytobrush, SpyGlass direct visual impression or SpyBite at the First Affiliated Hospital of Nanchang University between January 2010 and December 2019. Follow-up of the patients after ERCP was conducted by outpatient visits, telephone calls, or re-admission to the hospital for a follow-up period of at least 12 months or when the patients die. The study was approved by the ethics committee of the First Affiliated Hospital of Nanchang University and has therefore been performed in accordance with the ethical standards laid down in the 1964 Declaration of Helsinki and its later amendments (see Figure 1).

### Enrollment criteria

1. Age 18 or older;
2. Clinical diagnosis of indeterminate bile duct stricture;
3. No ERCP performance before;
4. All the patients signed informed consent form for ERCP procedure.

### Exclusion criteria

Loss of follow-up.

### Procedures

All the ERCP procedures were performed by experienced endoscopic physicians (each performed over 200 cases of ERCP per year). All the patients underwent ERCP after anesthesia with propofol. The ERCP procedures were performed with Olympus TJF-260v or JF-260v duodenoscope (Olympus, Japan). The SpyGlass Direct Visualization System equipment (Boston Scientific, United States) and accessories such as SpyBite mini-forceps (Boston Scientific, USA) were used for direct visual impression and cholangioscopy-guided tissue sampling of the bile duct, respectively. Cytobrush were performed with Cytomax II brush (Cook Medical, United States) and tissue biospy was performed with disposable biopsy forceps (Micro-Tech Endoscopy, China). The follow-up of the patients after ERCP, which lasted at least 12 months or until the patients die, was conducted by outpatient visits, telephone calls, and re-admission to the hospital.

### Outcomes, diagnostic gold criteria, and definitions

The primary outcome is the diagnostic value of ERCP-guided biopsy, cytobrush, SpyGlass direct visual impression and SpyGlass-guided biospy compared with the patient final diagnose (malignant or benign). The diagnostic value was demonstrated by sensitivity, specificity, accuracy, positive predictive value, negative predictive value, missed diagnosis rate and misdiagnosis rate. The gold diagnostic criteria for BBSs is determined as follows, (1) Pathological examination of surgical specimens confirmed benign. (2) If surgical histology is not available, the imaging or ERCP image after 12-month-follow-up demonstrates that the stricture is relieved or stabilized, and there is no obvious malignant progression. The gold diagnostic criteria for MBSs is determined as follows, (1) Pathological examination of surgical specimens confirmed malignant. (2) If surgical histology is not available, the imaging or ERCP after 12-month-follow-up demonstrates that the lesions have malignant progression. The second outcomes are adverse events, including post-ERCP pancreatitis (PEP), biliary infection, bleeding and perforation, whose definition is determinated according to European Society of Gastrointestinal Endoscopy (ESGE) Guideline (12). Correlated statistics were calculated as follows: If a represents true positive, b represents false positive, c represents false negative and d represents true negative, then sensitivity = a/(a + c); specificity = d/(b + d); positive predict value = a/(a + b); negative predict value = d/(c + d); accuracy = (a + d)/(a + b + c + d); missed diagnosis rate = c/(a + c) and misdiagnosis rate = b/(b + d).

### Statistics

Data were statistically analyzed using SPSS 25.0, and the Kolmogorov–Smirnov test was used to assess normality and homogeneity of all variables. Data for continuous normally distributed variables were expressed as mean ± standard deviation (M ± SD). Comparisons between groups were performed by one-way analysis of variance (ANOVA) and pairwise comparisons among groups were performed by the LSD method. Non-normally distributed data were expressed using median and interquartile spacing M (25, 75%), and analyzed by nonparametric test (Kruskal-Wallis H test). Categorical count data were expressed as n (%) and compared by the chi-square test, and pairwise comparisons between groups were performed by Bonferroni’s corrected Z test. Results were considered statistically significant at *p* < 0.05.

## Results

### Patients’ characteristics

A total of 1,002 patients were finally enrolled, including 534 patients in the brush cytology group, 412 in the biopsy group, 31 in the SpyGlass-guided direct visual impression and 25 in the Spybite group. The patient demographic information, the section of bile duct stricture, the etiology of bile duct stricture and post-ERCP surgical operations of the four groups were summarized in Table 1. There were 573 males (57.2%) and 429 females (42.8%) in all the patients with mean age 61.30 ± 13.41 years old. Of all the 1,002 patients, 744 (74.3%) patients were diagnosed with malignant strictures, including bile duct carcinoma in 480 (47.9%), gallbladder carcinoma in 31 (3.1%), pancreas carcinoma in 121 (12.1%) and duodenal papilla carcinoma in 112 (11.2%), and 258 (25.7%) patients were diagnosed with benign strictures. The final diagnosis was confirmed by surgical procedures after ERCP in 171 (17.1%) and by long-term (≥12 months) follow-up in 831 (82.9%).

**Table 1:** Patients general information.

	Cytobrush	Standard biopsy	Visual impression	Spybite	*p* value
Age (years)	61.43 ± 13.46	61.60 ± 13.14	58.52 ± 14.38	56.96 ± 15.21	0.373
Male	57.3% (306/534)	57.3% (236/412)	54.8% (17/31)	56.0% (14/25)	0.993
Strictures					
Distal	35.3% (188)^a^	84.0% (346)^b^	32.3% (10)^a^	32.0% (8)^a^	<0.001
Middle	18.6% (99)^a^	5.8% (24)^b^	6.5% (2)^ab^	8.0% (2)^ab^	<0.001
Proximal	46.1% (245)^a^	10.2% (42)^b^	61.3% (19)^a^	60.0% (15)^a^	<0.001
Cause of stricture					
Malignant	82.2% (439)^a^	67.5% (278)^b^	48.4% (15)^c^	48.0% (12)^c^	<0.001
Bile Duct Cancer	62.8% (334)^a^	28.9% (119)^b^	48.4% (15)^ac^	48.0% (12)^ac^	<0.001
Gallbladder Cancer	5.1% (27)^a^	1.0% (4)^b^	0^ab^	0^ab^	0.002
Pancreatic Cancer	13.0% (69)^a^	12.6% (52)^a^	0^b^	0^ab^	0.043
Papillary Cancer	1.7% (9)^a^	25.0% (103)^b^	0^a^	0^a^	<0.001
Benign	17.8% (95)^a^	32.5% (134)^b^	51.6% (16)^c^	52.0% (13)^c^	<0.001
Surgical Operations after ERCP	15.0% (80)	18.7% (77)	22.6% (7)	28.0% (7)	0.159

If *p* ≥ 0.05, no statistical difference was found among groups and no pairwise comparisons among groups were need. If p < 0.05, pairwise comparisons among groups were performed. Groups with the same superscript letter are not significantly different from each other. Conversely, Groups with completely different superscript letters are significantly different from each other.

The ERCP procedure parameters including bougienage/balloon dilation, endoscopic sphincterotomy (EST), precut, times of entrance of guide wire into pancreatic duct and placement of pancreatic duct stent were summarized in Table 2. Of all the patients, bougienage/balloon dilation was performed in 163 (16.3%), EST in 458 (45.7%), precut in 26 (2.6%), entrance of guide wire into pancreatic duct in 183(18.3%) and placement of pancreatic duct stent in 147(14.7%).

**Table 2:** Endoscopic procedures.

	Cytobrush	Standard biopsy	Visual impression	Spybite	*P*
Bougienage/balloon dilation	18.0% (96)	13.1% (54)	19.4% (6)	28.0% (7)	0.075
EST	48.5% (259)^a^	40.3% (166)^b^	61.3%(19)^a^	56.0% (14)^ab^	0.014
Precut	3.0% (16)	2.4% (10)	0	0	0.598
Entrance of guide wire into pancreatic duct	18.2% (97/534)	18.2% (75/412)	19.4% (6/31)	20.0% (5/25)	0.994
once	6.6% (35)	6.6% (27)	9.7% (3)	12.0% (3)	0.667
twice	7.9% (42)	6.3% (26)	6.5% (2)	4.0% (1)	0.741
≥3 times	3.7% (20)	5.3% (22)	3.2% (1)	4.0% (1)	0.677
Placement of pancreatic duct stent	15.7% (84)	13.6% (56)	12.90% (4)	12% (3)	0.782

If *p* ≥ 0.05, no statistical difference was found among groups and no pairwise comparisons among groups were need. If *p* < 0.05, pairwise comparisons among groups were performed. Groups with the same superscript letter are not significantly different from each other. Conversely, Groups with completely different superscript letters are significantly different from each other.

### Outcomes

The diagnostic results based on brush cytology, standard biopsy, visual impression or SpyBite were summarized, respectively, in Table 3. As far as sensitivity was concerned, standard biopsy group (48.6%) and SpyBite group (61.5%) were significantly higher than cytobrush group (32.0%), and visual impression group (100%) was significantly higher than any other group. There was no statistical difference between standard biopsy group and SpyBite group in sensitivity. As far as specificity was concerned, cytobrush group (99.0%), standard biopsy group (99.3%) and the SpyBite group (100%) were significantly higher than visual impression (55.6%), but there was no statistical difference among the three groups above. As far as accuracy was concerned, standard biopsy group (65.3%), and SpyBite group (80.0%) were significantly higher than cytobrush group (44.4%), and SpyBite group was significantly higher than visual impression group. The pairwise comparisons of positive predictive value and misdiagnosis rate among groups had the same results as that of specificity. The pairwise comparisons of negative predictive value and missed diagnosis rate among groups had similar results to that of sensitivity (Table 4).

**Table 3:** Diagnostic results.

Diagnosis based on brush cytology	Final diagnosis		Diagnosis based on standard biopsy	Final diagnosis	
	Malignant	Benign		Malignant	Benign
Malignant	139	1	Malignant	134	1
Benign	296	98	Benign	142	135

Diagnosis based on visual impression	Final diagnosis		Diagnosis based on SpyBite	Final diagnosis	
	Malignant	Benign		Malignant	Benign
Malignant	12	4	Malignant	8	0
Benign	0	5	Benign	5	12
Indeterminate	4	6			

**Table 4:** Main outcomes.

	Cytobrush	Standard biopsy	Visual impression	SpyBite	*p* value
Sensitivity (%)	32.0^a^	48.6^b^	100^c^	61.5^b^	<0.001
Specificity (%)	99.0^a^	99.3^a^	55.6^b^	100.0^a^	<0.001
Accuracy (%)	44.4^a^	65.3^bc^	54.8^ac^	80.0^bd^	<0.001
Positive predictive value (%)	99.3^a^	99.3^a^	75.0^b^	100.0^a^	<0.001
Negative predictive value (%)	24.9^a^	48.7^b^	100.0^c^	70.6^bc^	<0.001
Missed diagnosis rate (%)	68.0^a^	51.4^b^	0.0^c^	38.5^b^	<0.001
Misdiagnosis rate (%)	1.0^a^	0.7^a^	44.4^b^	0^a^	<0.001

If *p* ≥ 0.05, no statistical difference was found among groups and no pairwise comparisons among groups were need. If *p* < 0.05, pairwise comparisons among groups were performed. Groups with the same superscript letter are not significantly different from each other. Conversely, Groups with completely different superscript letters are significantly different from each other.

To sum up, conventional endoscopic sampling methods including cytobrush and standard biopsy had relatively low sensitivity and accuracy for differential diagnose between MBSs and BBSs although both of them had high specificity, which indicated that neither of them was good enough for early diagnosis of MBSs. Visual impression group had the highest sensitivity, the lowest specificity and relatively low accuracy among the four groups. SpyBite group had moderately high sensitivity and accuracy and extremely high specificity among the four groups. By combing the advantage of visual impression and SpyBite, we might get a best result for sensitivity, specificity and accuracy.

### Adverse events

The adverse events of ERCP were summarized in Table 5. The visual impression group and SpyBite group had significantly higher incidence of post-ERCP cholangitis than cytobrush group and standard biopsy group. There was no significant difference in the incidence of PEP, post-ERCP bleeding, perforation and ERCP-related deaths among the four groups (Table 5).

**Table 5:** Adverse events.

	Cytobrush	Standard biopsy	Visual impression	SpyBite	*p* value
Adverse events	22.8% (122/534)^a^	16.3% (67/412)^b^	41.9% (13/31)^c^	52.0% (13/25)^c^	<0.001
PEP	14.0% (75)	10.0%(41)	16.1% (5)	20.0% (5)	0.155
Post-ERCP cholangitis	6.9% (37)^a^	4.4% (18)^a^	19.4% (6)^b^	24.0% (6)^b^	<0.001
Post-ERCP cholecystitis	0	0.5% (2)	0	0	0.412
Post-ERCP bleeding	1.5% (8)	1.0% (4)	3.2% (1)	4.0% (1)	0.464
Perforation	0	0	0	0	–
ERCP-related deaths	0.4% (2)	0.5% (2)	0	0	0.958

PEP, post-ERCP pancreatitis. If *p* ≥ 0.05, no statistical difference was found among groups and no pairwise comparisons among groups were need. If *p* < 0.05, pairwise comparisons among groups were performed. Groups with the same superscript letter are not significantly different from each other. Conversely, Groups with completely different superscript letters are significantly different from each other.

## Discussion

Biliary strictures are classified as benign strictures and malignant strictures. The causes of benign biliary strictures include iatrogenic, inflammatory, autoimmune or infectious factors, of which the most common one is iatrogenic, including bile duct injury during liver transplantation or cholecystectomy (13). The most common causes of MBSs are bile duct cancer and pancreatic cancer (14), which was also confirmed in our study.

Not only the prognosis but also the management of benign and malignant bile duct strictures varies. The preferred treatment for benign biliary strictures is the placement of biliary stent by ERCP, whereas the best treatment for malignant biliary strictures is surgery (1). Furthermore, endoscopic biliary stent placement and drainage is currently considered the best palliative treatment for MBSs that cannot be removed surgically, and preoperative biliary stent placement is also recommended to relieve biliary obstruction in patients who have cholangitis (1). The results of our study showed that about 20% of the patients who underwent surgery were ultimately proven to have BBSs, suggesting that these patients should not had accepted surgical treatment. On the other hand, MBSs that are not confirmed promptly might have a high metastatic potential so that the patients might lose the opportunity of surgery and hence have a poor prognosis. Therefore, the differential diagnosis of benign and malignant strictures is crucial before therapeutic approaches are implemented.

By analyzing the patients’ general information, we found that the proportion of patients with distal strictures in biopsy group was significantly higher than that of proximal and middle strictures. The possible reason might be that biopsy by forceps was easier to perform in distal strictures. Our study also showed a higher ratio of balloon dilatation was performed in the SpyGlass Group, which might be due to the thicker caliber of SpyGlass, and bile duct dilatation facilitated the insertion of SpyGlass.

A previous study showed that the specificity of both cytobrush and biopsy was high enough, reaching more than 99%, but the sensitivity was low, with neither exceeding 50% (15). The low sensitivity of cytobrush might have several reasons. Firstly, the amount of cells obtained by cytobrush is often not enough for positive results. Secondly, biliary tract tumors are mostly well differentiated or moderately differentiated adenocarcinoma, with higher differentiation in surface cells than deeper cells. Cell brushes can only obtain cells on the surface of biliary tract tumors, resulting in false negative results (6). The results of our study were similar to those of the previous studies, and further analysis of our study demonstrated that the standard biopsy was better than the cytobrush in terms of sensitivity, accuracy, and negative predict value, which might be related to the larger amount of tissue taken by biopsy than that by cytobrush. However, the sensitivity of biopsy might also be negatively affected by inaccurate biopsy site guided only by X-ray, especially for proximal bile duct stricture.

A previous meta-analysis that included six studies with a total of 283 patients showed that the combined sensitivity and specificity of Spyglass-guided visual impression for MBSs was 94 and 95%, respectively, (16). Our study also showed higher sensitivity of Spyglass-guided visual impression compared with cytobrush and standard biopsy. The diagnosis accuracy in visual impression group seemed to be higher than that in cytobrush group and standard biopsy group, but was only statistically higher than that in cytobrush group. The specificity of SpyGlass-guided visual impression in our study was lower than that reported in the literature, probably because most of the patients in our study who underwent SpyGlass examination had a high clinical suspicion of malignant strictures, and the judgment by some endoscopists during SpyGlass examination was influenced by the patient’s medical history and tended to make a diagnosis of malignancy, resulting in high false positive rate. Moreover, the criteria for endoscopic manifestations of benign and malignant strictures by visual impression have not yet been standardized, and the diagnosis made by endoscopists might be subjective. Some cohort studies and meta-analyses have reported that SpyBite is superior to ERCP-guided bile duct biopsy and brushing in terms of diagnostic yield and potential cost-effectiveness (8). Our study showed that the specificity and sensitivity of Spybite were similar to those reported but were not statistically different from those of cytobrush and biopsy, while the accuracy of SpyBite was significantly higher than that of cytobrush, although had no statistical difference from that of standard biopsy. The higher sensitivity and accuracy of SpyGlass-related technique for the diagnosis of indeterminate bile duct stricture is due to accurate positioning of the lesion site under direct vision of SpyGlass and the enough tissues obtained by SpyBite forceps. It should be noted that the sample taken by SpyBite was relatively small, which might be easily lost in the process of tissue slicing and affect the pathologist’s judgment, so endoscopists should try to take samples as many as possible and discuss with pathologists to further improve the diagnosis sensitivity of SpyBite.

PEP is the most common adverse event after ERCP, and the incidence of PEP has been reported to be 3.5–9.7% (12). Some studies have shown that endoscopic placement of covered self-expandable metal stents and bile duct dilatation are risk factors for PEP (12, 17). The incidence of PEP in our study was higher compared with that of previous studies, which might be due to the high ratio of biliary duct dilatation and the placement of covered self-expandable metal stents. However, the results of our study showed no significant difference in PEP between different endoscopic diagnostic methods. Interestingly, our study showed that the incidence of cholangitis in SpyGlass-guided visual impression group and SpyBite group was higher than that of cytobrush and standard biopsy group, probably because SpyGlass performance required water injection in the bile duct to make a clear vision, which might lead to retrograde bile duct infection. All the patients suffered from post-SpyGlass cholangitits recovered soon after timely usage of antibiotics without severe clinical outcomes. Interestingly, all the patients with adequate biliary drainage such as stent placement in the SpyGlass-guided visual impression group and the SpyBite group avoided cholangitis and the usage of antibiotics. Therefore, we believed post-SpyGlass cholangitits could be controlled by antibiotics, and might be avoided by adequate biliary drainage.

In conclusion, our study showed that SpyBite combined with SpyGlass-guided visual impression was better for differential diagnosis of benign and malignant bile duct strictures in terms of sensitivity and accuracy compared with conventional endoscopic diagnostic methods such as cytobrush and forceps biopsy. Furthermore, the incidence of adverse events after SpyGlass examination were similar to those after conventional endoscopic diagnostic methods except for higher cholangitis rate.

Most of the previous literature had not clearly elaborated the diagnostic criteria for benign and malignant biliary strictures, while our study determinated the diagnostic criteria for benign and malignant strictures according to the latest guideline consensus (1, 17), which ensured the reliability of the differential diagnosis of biliary strictures. However, our study was single-central and retrospective, and therefore also had limitations. Firstly, the number of SpyGlass-guided visual impression and SpyBite cases included in our study was small, which was a possible reason why some of the results were not statistically different. Secondly, the disparity in numbers between different groups might reduce statistical power. We have considered sampling method and paired test when we designed the study, but this might cause a new error called sampling error. Besides, sampling and paired test will inevitably reduce the sample size of each group, thus increasing the sample representative bias. On the other hand, although the disparity in numbers between different groups might reduce statistical power, it has much lesser effect on the positve statistical results because *p*-value was calculated according to two parameters including statistics and degrees of freedom, both of which have taken sample size into consideration. Actually, a small sample size is more difficult to obtain statistical significance. Therefore, after weighing the advantages and disadvantages, we maintained the statistical analysis of different groups with disparity in numbers. More multi-central randomized clinical trials are needed in the future to further explore the value of different endoscopic diagnostic methods in the differential diagnosis of benign and malignant biliary strictures.

## Data availability statement

The raw data supporting the conclusions of this article will be made available by the authors, without undue reservation.

## Ethics statement

The studies involving human participants were reviewed and approved by Ethics Committee of the First Affiliated Hospital of Nanchang University. The patients/participants provided their written informed consent to participate in this study.

## Author contributions

LZ collected and analyzed the data, interpreted the results, and wrote the manuscript. Z-QH, Z-WW, J-BH, Z-ZY, X-PY, Z-PY, R-LC, and J-LH participated in the follow-up of the patients, and collection and analysis of the data. Y-XC designed the study, supervised the project and revised the manuscript. All authors contributed to the article and approved the submitted version.

## Funding

This study was supported by National Natural Science Foundation of China (No. 82160694), the project of the Jiangxi Provincial Department of Science and Technology (No. 20202BBGL73109), the project of the Health and Family Planning Commission of Jiangxi Province (No. 20195082), the project of Jiangxi Clinical Research Center for Gastroenterology (No. 20201ZDG02007), and Jiangxi Postgraduate Innovation Special Fund Project (No. YC2021-S192).

## Conflict of interest

The authors declare that the research was conducted in the absence of any commercial or financial relationships that could be construed as a potential conflict of interest.

## Publisher’s note

All claims expressed in this article are solely those of the authors and do not necessarily represent those of their affiliated organizations, or those of the publisher, the editors and the reviewers. Any product that may be evaluated in this article, or claim that may be made by its manufacturer, is not guaranteed or endorsed by the publisher.
